# Velocity Gradient Separation Reveals a New Extracellular Vesicle Population Enriched in miR-155 and Mitochondrial DNA

**DOI:** 10.3390/pathogens10050526

**Published:** 2021-04-27

**Authors:** Myriam Vaillancourt, Audrey Hubert, Caroline Subra, Julien Boucher, Wilfried Wenceslas Bazié, Julien Vitry, Sofiane Berrazouane, Jean-Pierre Routy, Sylvie Trottier, Cécile Tremblay, Mohammad-Ali Jenabian, Abderrahim Benmoussa, Patrick Provost, Philippe A. Tessier, Caroline Gilbert

**Affiliations:** 1 Centre de Recherche du CHU de Québec-Université Laval, T1-49, 2705 boulevard Laurier, Québec, QC G1V 4G2, Canada; myriam.vaillancourt@crchudequebec.ulaval.ca (M.V.); audrey_hubert15@hotmail.com (A.H.); caro_subra@yahoo.fr (C.S.); julien.boucher.2@ulaval.ca (J.B.); wilfried-wenceslas.bazie.1@ulaval.ca (W.W.B.); Julien.Vitry@crchudequebec.ulaval.ca (J.V.); Sofiane.Berrazouane@crchudequebec.ulaval.ca (S.B.); sylvie.trottier@crchudequebec.ulaval.ca (S.T.); rahimbenmoussa@gmail.com (A.B.); patrick.provost@crchudequebec.ulaval.ca (P.P.); philippe.tessier@crchudequebec.ulaval.ca (P.A.T.); 2 The Henry M. Jackson Foundation for the Advancement of Military Medicine, Bethesda, MD 20817, USA; 3 U.S. Military HIV Research Program, Walter Reed Army Institute of Research, Silver Spring, MD 20910, USA; 4 Programme de Recherche sur les Maladies Infectieuses, Centre Muraz, Institut National de Santé Publique, Bobo-Dioulasso 01 BP 390, Burkina Faso; 5 Chronic Viral Illness Service and Division of Hematology, McGill University Health Centre, Montréal, QC H4A 3J1, Canada; jean-pierre.routy@mcgill.ca; 6 Infectious Diseases and Immunity in Global Health Program, Research Institute, McGill University Health Centre, Montréal, QC H4A 3J1, Canada; 7 Centre de Recherche du CHU de Québec, Department of Microbiology, Infectiology and Immunology, Faculty of Medicine, Université Laval, T1-49, 2705 boulevard Laurier, Québec, QC G1V 4G2, Canada; 8 Centre de Recherche du Centre Hospitalier de l’Université de Montréal, Montréal, QC H3C 3J7, Canada; c.tremblay@umontreal.ca; 9 Département de Microbiologie, Infectiologie et Immunologie, Faculté de Médecine, Université de Montréal, Montréal, QC H3T 1J4, Canada; 10 Département des Sciences Biologiques et Centre de Recherche CERMO-FC, Université du Québec à Montréal (UQAM), Montréal, QC H2L 2C4, Canada; jenabian.mohammad-ali@uqam.ca; 11 Department of Nutrition, CHU Sainte-Justine—Université de Montréal, Montréal, QC H3T 1J4, Canada

**Keywords:** biomarker, calprotectin, extracellular vesicles, HIV, miR-92, miR-155, miR-223, mtDNA, velocity gradient, virions

## Abstract

Extracellular vesicles (EVs) and their contents (proteins, lipids, messenger RNA, microRNA, and DNA) are viewed as intercellular signals, cell-transforming agents, and shelters for viruses that allow both diagnostic and therapeutic interventions. EVs circulating in the blood of individuals infected with human immunodeficiency virus (HIV-1) may provide insights into pathogenesis, inflammation, and disease progression. However, distinguishing plasma membrane EVs from exosomes, exomeres, apoptotic bodies, virions, and contaminating proteins remains challenging. We aimed at comparing sucrose and iodixanol density and velocity gradients along with commercial kits as a means of separating EVs from HIV particles and contaminating protein like calprotectin; and thereby evaluating the suitability of current plasma EVs analysis techniques for identifying new biomarkers of HIV-1 immune activation. Multiple analysis have been performed on HIV-1 infected cell lines, plasma from HIV-1 patients, or plasma from HIV-negative individuals spiked with HIV-1. Commercial kits, the differential centrifugation and density or velocity gradients to precipitate and separate HIV, EVs, and proteins such as calprotectin, have been used. EVs, virions, and contaminating proteins were characterized using Western blot, ELISA, RT-PCR, hydrodynamic size measurement, and enzymatic assay. Conversely to iodixanol density or velocity gradient, protein and virions co-sedimented in the same fractions of the sucrose density gradient than AChE-positive EVs. Iodixanol velocity gradient provided the optimal separation of EVs from viruses and free proteins in culture supernatants and plasma samples from a person living with HIV (PLWH) or a control and revealed a new population of large EVs enriched in microRNA miR-155 and mitochondrial DNA. Although EVs and their contents provide helpful information about several key events in HIV-1 pathogenesis, their purification and extensive characterization by velocity gradient must be investigated thoroughly before further use as biomarkers. By revealing a new population of EVs enriched in miR-155 and mitochondrial DNA, this study paves a way to increase our understanding of HIV-1 pathogenesis.

## 1. Introduction

Extracellular vesicles (EVs) are heterogeneous groups of vesicles found in most biological fluids. EVs from blood cells were first visualized and described over 50 years ago [1,2]. Nowadays, EVs are considered as theranostic tools used for both diagnosis and therapeutic purposes. Changes in EVs abundance and contents have been correlated with conditions such as preeclampsia [3], cancers [4], and HIV infection [5,6]. Moreover, their role in cell-to-cell communication has been established as they are released from donor cells into the medium transferring their components to recipient cells. These components include microRNAs [7], signaling, and apoptotic proteins from the cells of origin and sometimes viral proteins and genetic material [5,8,9,10,11]. Based on their biogenesis, release pathways, size, content, and functions, EVs are classified into different subsets; namely exosomes, microvesicles (MVs), and apoptotic bodies (ApoBDs). Their characterizations and functions seem to be essential but challenging fields of study in many research areas. Indeed, the heterogeneity of EVs [12], their purification, and analytical methods, along with their physical similarity to virions, have made studies difficult to interpret. Founded in 2012, the International Society for Extracellular Vesicles (ISEV) has contributed significantly to establishing guidelines in this new research field and has helped with classification but many challenges remain to address when it comes to their distinction from viral particles [13]. Herein, we address these issues in the analysis of EVs from plasma in the context of HIV-1 infection.

In HIV-1 patients, EVs present abnormalities in terms of numbers, size, and contents [5,6]. Their abundance and size are correlated with the CD4/CD8 ratio [5,6], a marker of disease progression. Some EVs contain more Nef protein, responsible for the degradation of CD4 receptors [8] and pro-apoptotic effects on CD4 T cells [14,15]. These EVs may also have a proinflammatory effect by transporting disintegrin ADAM17, a metalloprotease that produces active TNF by cleaving the inactive form on surrounding cells [16]. The complete HIV-1 RNA transcript, including TAR, can be found in EVs [17], which may transfer this RNA by fusion with uninfected CD4 T cells, making the latter more susceptible to infection [11], or stimulating the production of proinflammatory cytokines in the case of macrophages [18]. Studies have shown that EVs in HIV-1 patients contain the microRNA miR-155, which regulates the expression of genes involved in homeostasis and the immune response [5,6,19]. These data have led to consider EVs as potential biomarkers for monitoring pathogenesis associated with infection and inflammatory status [5].

EVs are categorized according to their tissue of origin, role in intercellular communication, size, and density [13]. Plasma-membrane-derived vesicles of 100 nm in diameter usually have densities below 1.1 g/mL, whereas endosome-derived vesicles, known as exosomes are smaller (30–100 nm in diameter) and denser, around 1.13–1.19 g/mL [13]. The density of larger vesicles like apoptotic bodies ranges from 1.18–1.28 g/mL in the case of apoptotic bodies to 1.25–1.30 g/mL for so-called microparticles or large EVs [13].

Over the past decade, technological advances have allowed the characterization of exosomes formed in late endocytic compartments and released into the extracellular medium by fusion of multivesicular bodies with the plasma membrane. They were first described as a mechanism of recycling specific proteins such as the transferrin receptor in maturing reticulocytes [20,21]. Endosomal membrane activities, such as acetylcholinesterase (AChE), have also been associated with exosomes [20]. Among the proteins now recognized as more abundant in exosomes are Alix and tumor susceptibility gene 101 (Tsg101), linked to the endosomal sorting complex required for transport (ESCRT). Tetraspanins CD9, CD63, and CD81 are also known as exosome markers [13]. More recently, small particles enriched in heat shock proteins such as Hsp70/90, α-2,6-sialyltransferase 1 (ST6Gal-I), and amphiregulin have been described and given the name exomeres [22,23,24].

The subcellular vesicle fraction of plasma thus contains EVs, some of which may be apoptotic, exosomes, exomeres, micro-particulate complexes bearing high-density lipoprotein (HDL), immunoglobulin, calprotectin, miRNA, RNA, and DNA, and may contain virions. Human cell culture supernatant may also contain any, or all, of the above. The recovery and characterization of EVs remain a daunting challenge despite recent technological progress. EVs purification methods are classified according to their enrichment factor and specificity [14]. Differential centrifugation provides enrichment with moderate specificity, that is, EVs mixed with various amounts of free proteins and virions [13]. Several commercial kits offer rapid alternatives to stepwise ultracentrifugation [25,26]. However, separation from proteinaceous materials such as serum albumin, calprotectin, or immunoglobulins remains problematic [27,28,29,30]. Treatment with proteinase K [5,31] and steric exclusion chromatography [30,32] are somewhat useful. Combinations of low-level extraction, microfiltration, and steric exclusion chromatography have been used to purify single EVs subpopulations [13]. Immunocapture targeting surface markers can be used to obtain specific EVs-enriched fraction [28]. Subsequent discrimination between EVs subpopulations requires centrifuging the EVs pellet on a sucrose or iodixanol density gradient [33].

Separating EVs from HIV-1 virions has been particularly challenging since these particles are very similar in size, density, as well as lipid, sugar, and protein composition [34]. Indeed, virions can be shed by mechanisms similar to exosome release, that is, through multivesicular bodies and the ESCRT apparatus [35], thus acquiring surface proteins such as host CD63 and CD81 tetraspanins, MHC-II, Tsg-101, and ICAM-1 [36]. Viral proteins such as Gag, Nef, Env, and Vpu, as well as viral RNA can also be incorporated into EVs [35,36]. Separating and purifying methods need to be improved for plasma to validate the EVs or protein co-precipitated with theirs as biomarkers of HIV-1 pathogenesis. EVs have been separated from virions by immunocapture using an antibody targeting AChE-E [10,37] or by two-step chromatography using steric exclusion followed by heparin affinity [38], heparin being selective for viral particles. The resulting fraction can be resolved on an iodixanol velocity gradient [37,39]. In this study, we compared the effectiveness of the sucrose density gradient and iodixanol density or velocity gradients at separating EVs from HIV-1 and other vesicles and proteins in precipitates obtained from plasma using a commercial kit. We found that EVs potentially characterizing HIV-1 infection were heterogeneous. The iodixanol velocity gradient was best suited for its resolution and the separation of contaminating proteins, such as calprotectin. We also identified a new type of EVs, preferentially enriched in miR-155 and mitochondrial DNA.

## 2. Results

### 2.1. Iodixanol Velocity Gradient Separates EVs from HIV-1 Virus Particles from Cell Culture Supernatant

**Figure 1:**
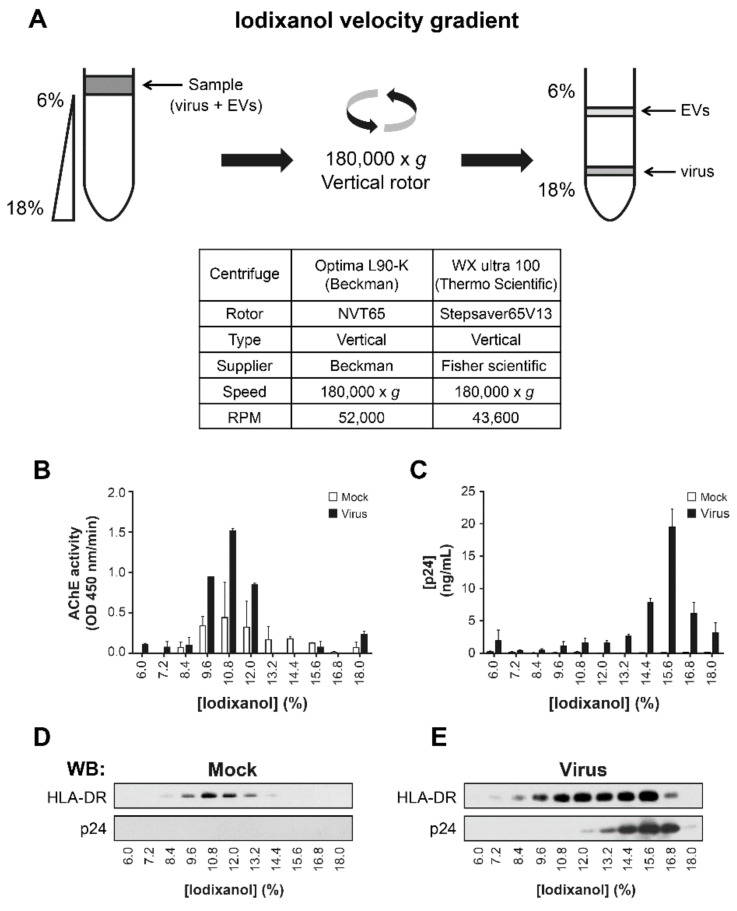
Iodixanol velocity gradient separates HIV-1 from EVs in infected cell-lines. In a continuous 6–18% iodixanol gradient (Optiprep^TM^), the sample is laid on the top of the gradient and ultracentrifuged in a vertical rotor for 50–75 min (**A**). Raji-CD4 cells were cultured with NL4-3 virus or mock for five days. Microfiltered supernatant was centrifuged at 100,000× *g* for 45 min. The pellet was re-suspended in PBS and laid on an iodixanol velocity gradient and centrifuged for 50 min. The relative abundance of EVs based on AChE activity (**B**) and the abundance of the virus based on p24 ELISA (**C**) were assessed. EVs from Raji-CD4 cells were recovered as described above and laid on iodixanol velocity gradient and centrifuged for 75 min. Precipitated proteins were probed for host membrane protein such as HLA-DR or viral protein p24 by western blots (**D**,**E**).

Due to the similarities between enveloped virus particles, like HIV-1, and EVs, the specificity of separating methods was firstly challenged with material from cell culture. We addressed the purification of EVs from Raji-CD4 cell culture supernatant, using ExoQuick-TC^TM^, sucrose density gradient, or iodixanol velocity gradient as shown in Figure 1 and Figure S1. Velocity gradient, obtained with 6–18% iodixanol and used by several groups [11,14,15,37,40,41], may be efficient in separating virions from EVs. In the iodixanol velocity gradient, the density ranged from 1.037 g/mL to 1.112 g/mL, and the time of centrifugation in the vertical rotor was short (Figure 1A). In this gradient, equilibrium is not reached since the density of virions is inferior to 1.16 g/mL [42]. Suspended particles migrate along the gradient in the function of their velocity rather than on their density. Consequently, EVs and HIV-1 did not reach the same fractions (Figure 1). AChE activity, which quantifies EVs abundance, was not found in the same fractions as HIV-1 p24 protein (Figure 1B,C). HLA-DR was found in the same fractions as AChE activity (Figure 1B,E). An amount of HLA-DR also co-fractionnated with p24 protein, reflecting a host-expression by the virus. AChE activity and Tsg-101 expression, another EVs marker, increased with the pelleted EVs after HIV-1 infection, as expected (Figure S1A,B). Exosome protein markers such as LAMP-2 and Tsg 101 and viral capsid protein p24 were concentrated in the ExoQuick-TC^TM^ pellet (Figure S1B). These results show that the ExoQuick-TC^TM^ kit does not separate the virus from EVs. With a density of about 1.16 g/mL [40,43], enveloped virus gravitates with EVs (1.15–1.19 g/mL [44]) to the 1.10–1.20 g/mL range (Figure S1C) in a sucrose density gradient. HLA-DR was seen in these p24-positive fractions (Figure S1D). As ExoQuick-TC^TM^ and sucrose density gradients do not seem to separate HIV-1 virions from EVs, we considered using an iodixanol velocity gradient.

**Figure 2:**
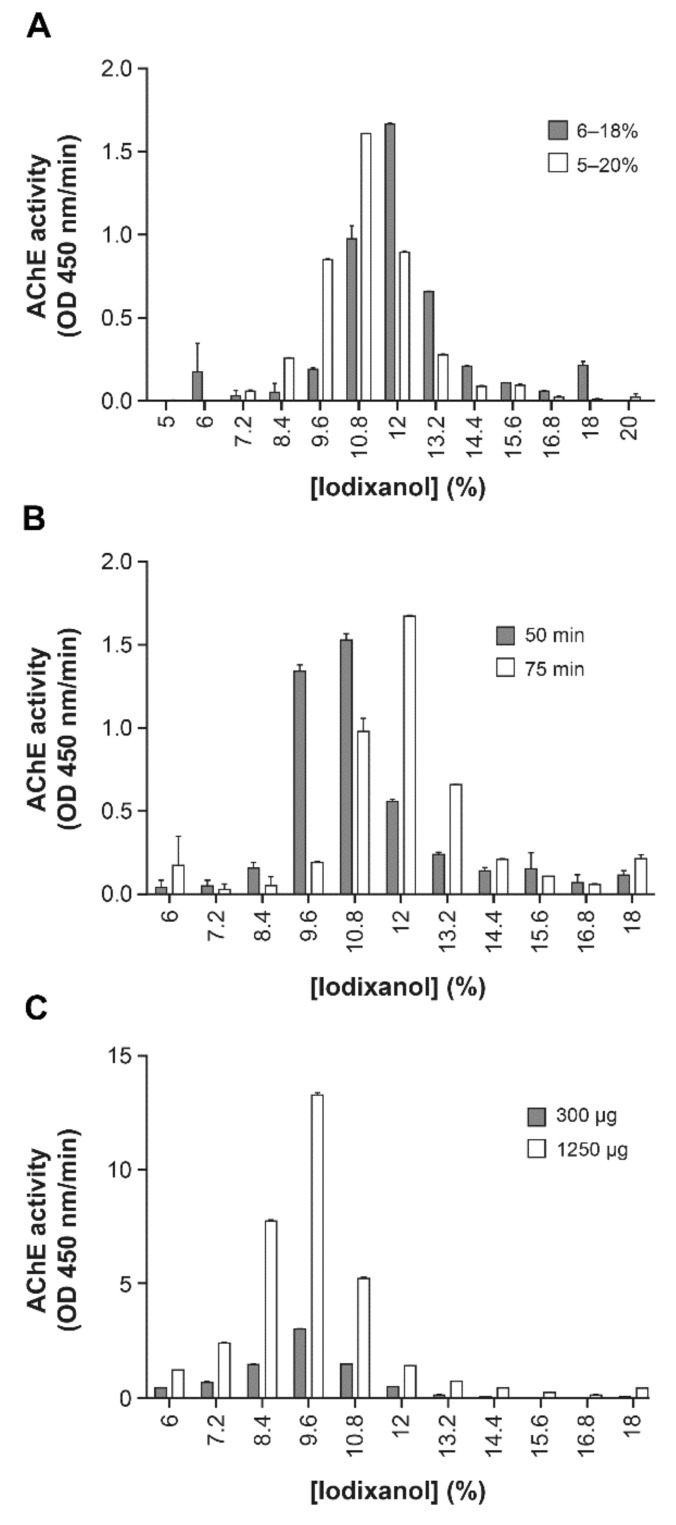
Centrifugation time and iodixanol concentration determine AChE-positive EVs migration through the velocity gradient. Raji-CD4 cells were cultured for five days. Microfiltered supernatant was centrifuged at 100,000× *g* for 45 min. The pellet was overlaid on iodixanol velocity gradients (6–18% or 5–20%) and centrifuged for 75 min (**A**). Iodixanol velocity gradients were centrifuged for 50 or 75 min (**B**). Different amounts of protein (300 µg or 1250 µg) were overlaid on gradients and centrifuged for 50 min (**C**). AChE activity is presumed to indicate a relative abundance of EVs.

This method also allowed confirmation of the previous finding that HIV-1 infection increases EVs production [41,45] and allows the separation of EVs and virions. The basal production of EVs by uninfected Raji-CD4 was next used to assess the impact of iodixanol concentration (Figure 2A), centrifugation time (Figure 2B), and protein overlay quantity (Figure 2C) on particle separation. The duration of centrifugation was the most sensitive parameter (Figure 2B) rather than the iodixanol concentration increase (Figure 2A). Protein concentration alone did not appear to influence migration (Figure 2C). Since the iodixanol velocity gradient is efficient to separate EVs from virions, this gradient might be best adapted for complex fluids such as plasma, particularly in the context of HIV-1 infection.

### 2.2. Optimizing Plasma EVs Purification by Adding HIV-1

#### 2.2.1. Commercial Kits

**Figure 3:**
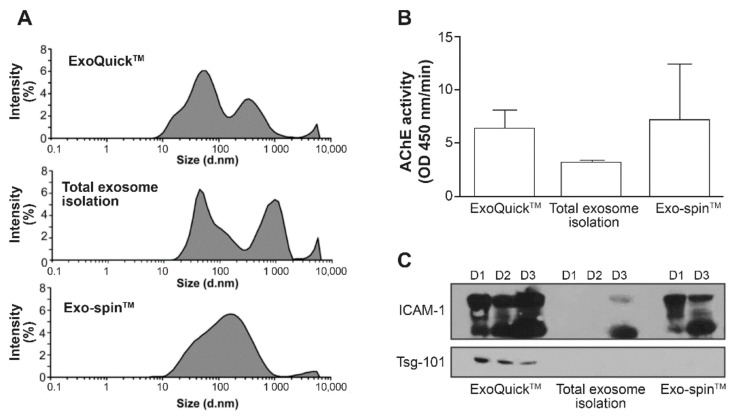
Purification of plasma EVs using commercial kits. EVs in plasma from HIV-negative individuals (D = donor) were precipitated using ExoQuick™ (SBI), the Total Exosome Isolation kit (Thermo scientific), and Exo-Spin™ (Cell Guidance Systems). Graphed average hydrodynamic size (DLS), 3 individuals, 2 measurements each (**A**); relative recovery of AChE EVs in precipitates (*n* = 3 for ExoQuick^TM^ and Total Exosome isolation kit, *n* = 2 for Exo-Spin^TM^) (**B**); and EVs-positive for cellular membrane markers (ICAM-1) or EVs marker (Tsg-101) by western blots (**C**).

The clinical relevance for detecting EVs in plasma has led companies to develop kits for purifying EVs in biological fluids. As seen in Figure S1, EVs and virions co-precipitated in samples from HIV-1-infected cells supernatants. To analyze the EVs composition in plasma from healthy individuals, we first compared ExoQuick^TM^ from System Biosciences, the Total Exosome Isolation kit from Thermo scientific and Exo-spin^TM^ from Cell Guidance Systems (Figure 3). The measurement of the hydrodynamic size of EVs by DLS shows a highly variable particle range (Figure 3A). Proteinase K treatment did not eliminate big particles or aggregates with the Total EVs isolation kit. Similar AChE activities were measured in EVs obtained using ExoQuick^TM^ and Exo-spin^TM^ (Figure 3B). AChE activity was lower in EVs obtained with the Total Exosome Isolation kit since proteinase K was used as recommended by the supplier. Western blots (Figure 3C) revealed that only ExoQuick^TM^ precipitated Tsg-101-bearing EVs from plasma of healthy individuals, confirming that this kit is somewhat selective for EVs despite proteins and HIV-1 being precipitated (Figure S2A).

#### 2.2.2. Calprotectin Separation

**Figure 4:**
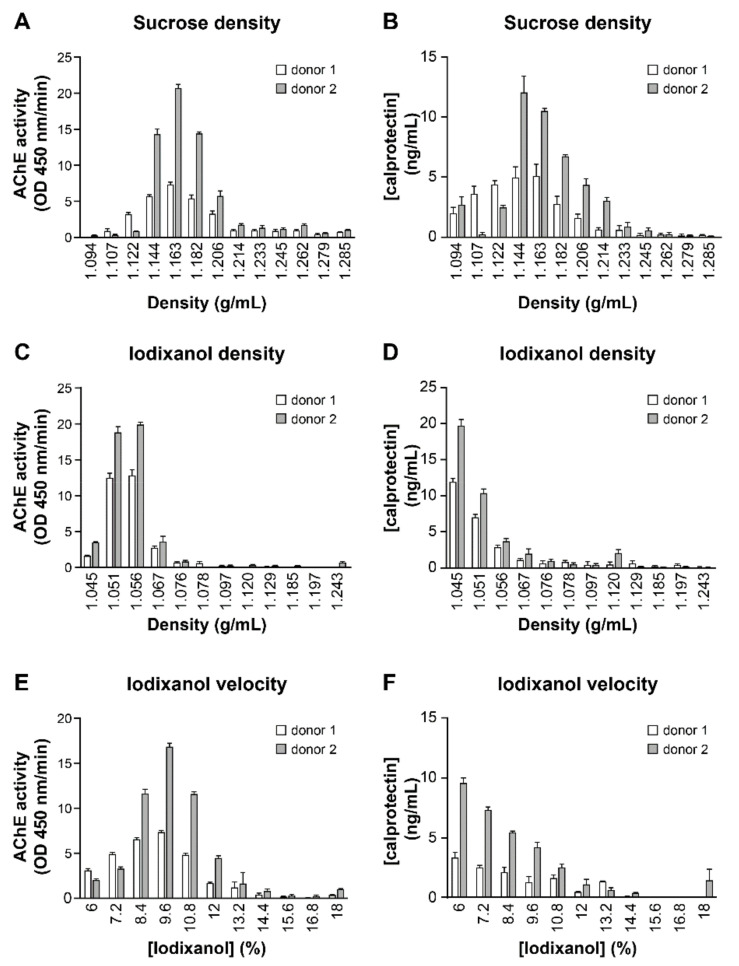
Separation of plasma EVs and calprotectin by density or velocity gradients. Plasma EVs from two HIV-1 negative individuals were precipitated by ExoQuick™ before overlaid on sucrose density (**A**,**B**), iodixanol density (**C**,**D**), or iodixanol velocity (**E**,**F**) gradients. AChE (left panels) and calprotectin (right panels) in each gradient fractions were respectively measured by enzymatic assay and ELISA.

Plasma contain abundant potential contaminants like microparticles (from platelets), immune complex aggregates, a significant amount of immunoglobulins (up to 10 mg/mL), albumin, and inflammatory protein such as calprotectin and all these components can interfere with EVs precipitation. Sucrose density (Figure 4A,B), iodixanol density (Figure 4C,D), and iodixanol velocity (Figure 4E,F) gradients were used to separate calprotectin from plasma AChE-positive EVs. The results show that velocity gradient (Figure 4E,F) fractions richer in AChE activity (8.4–10.8% iodixanol) are not enriched in calprotectin (highest in 6–8.4% iodixanol). This observation suggests that calprotectin is not bound to the AChE-EVs sub-types in contrast with results obtained using two density gradients (Figure 4A–D).

#### 2.2.3. Spiked Plasma with Virions

**Figure 5:**
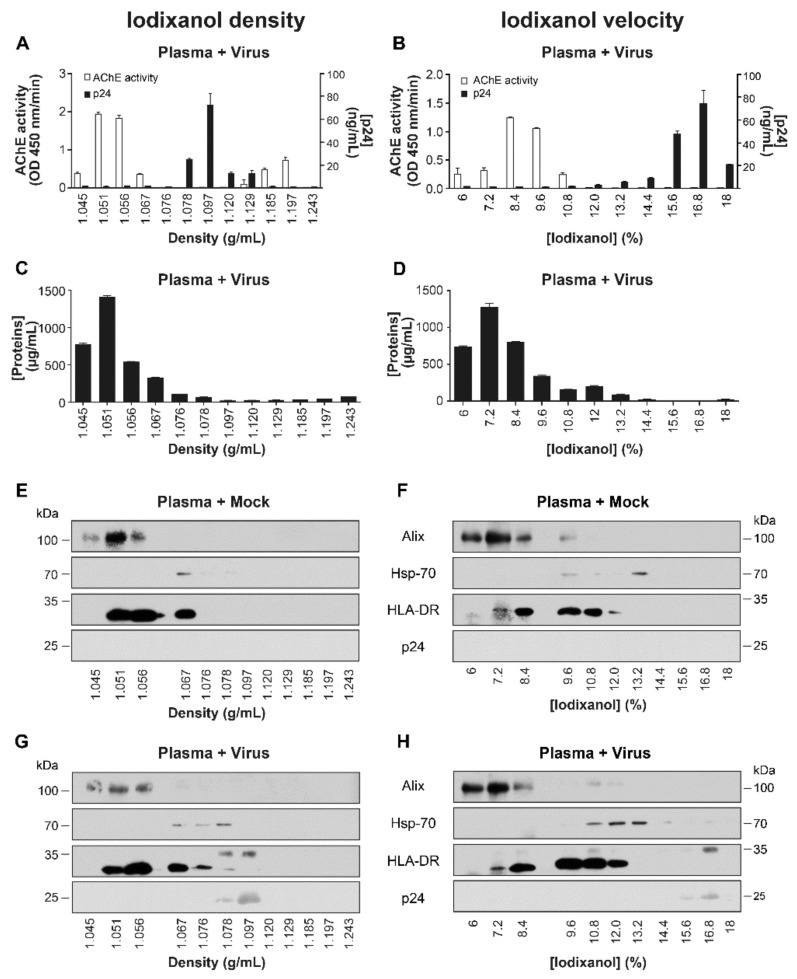
Separation of plasma proteins, EVs, and HIV-1 in the iodixanol density and velocity gradients. A pool from five plasma samples from HIV-1 negative individuals were spiked with virions or mock from Raji-CD4 infected-cells. Then EVs were precipitated by ExoQuick^TM^. The pellet was re-suspended in PBS and laid on iodixanol density (left panels) or velocity (right panels) gradients. Acetylcholinesterase (AChE) (**A**,**B**), p24 (**A**,**B**), total (**C**,**D**), and specific proteins (**E**–**H**) were quantified using an enzymatic assay, an ELISA, BCA kit, and western blot respectively.

To separate all types of EVs, including virions, we added virus recovered from Raji-CD4 HIV-1 or mock-infected cells to a pool of five plasma samples from HIV-1 negative individuals. After EVs precipiation with ExoQuick^TM^, EVs were overlaid onto three types of gradients. As seen in Figure S2B, virions (p24) and AChE activity were found in the same fractions in sucrose density gradients (Figure S2B), as opposed to iodixanol density (Figure 5A) and velocity (Figure 5B) gradients where they were found in different fractions. Both iodixanol gradients concentrated the proteins mostly in the first two or three fractions (Figure 5C,D), as shown for calprotectin (Figure 4D,F). The resolution was further characterized by precipitating proteins in each fraction and performing western blots (Figure 5E–H). Plasma spiked with virus contained all markers in larger amounts than the plasma spiked with mock-infected cells, regardless of gradient type. EVs marker Alix was found in the first three fractions of both gradients. Heat-shock protein (HSP) 70, of an endoplasmic reticulum origin and described as a stress marker in EVs called exomeres [22], was visible in the neighboring density gradient fraction as p24 (Figure 5G) but in different velocity gradient fractions (Figure 5H). The virions protein detected by western blot (Figure 5G,H) reflecting the p24 ELISA pattern (Figure 5A,B) and confirming that HIV-1 and some EV populations gravitated to the same fraction when the density gradient was used. In summary, we observed approximately the same marker distribution profile in both gradients, but the velocity gradient performed better at separating virions from Hsp-70-and HLA-DR bearing EVs. Figure 5 shows the heterogeneity of EVs in plasma and a better resolution of EVs from plasma and virions on velocity iodixanol gradients. The heterogeneity of EVs isolated from HIV-1 negative individual’s plasma was confirmed by the detection of Tsg-101 in the same fraction as AChE, the presence of Lamp-2 in fractions containing less AChE, and the heterogeneous distribution of soluble and membrane-bound forms of ICAM-1 all corroborate the heterogeneity of EVs (data not shown).

**Figure 6:**
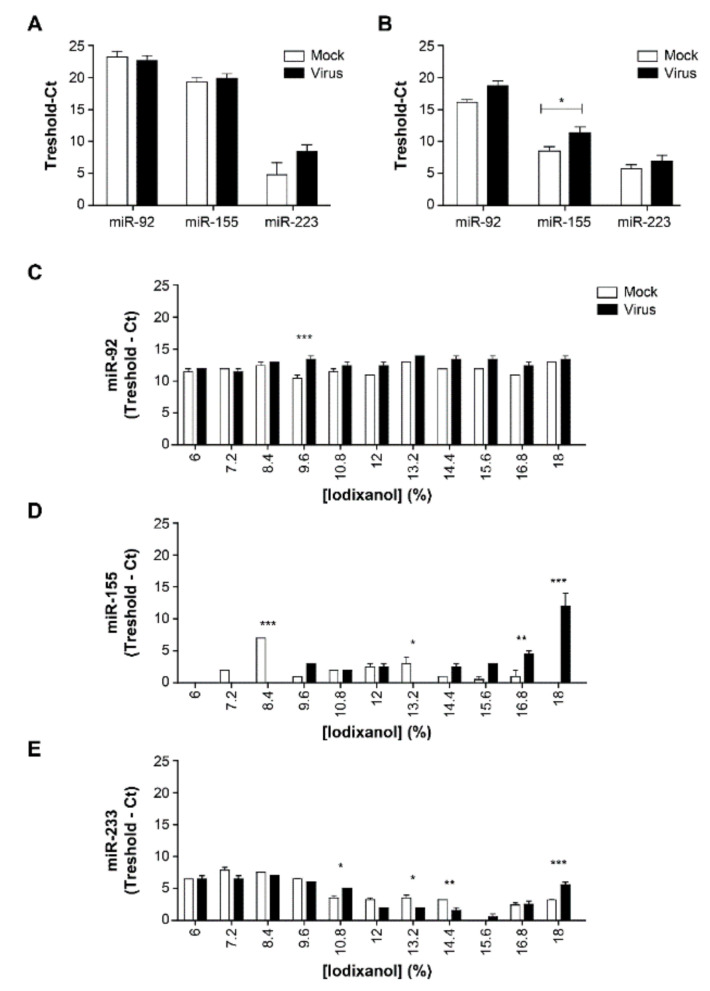
Comparison of HIV-1 infection and mock infection of Raji-CD4 cells in terms of EVs-borne miR-92, miR-155, and miR-223 distribution in the iodixanol velocity gradient. RT-PCR was used to measure microRNA in whole cells (**A**), or in a 100,000× *g* centrifugal pellet of the culture supernatant (**B**) of Raji-CD4 infected-cells. A pool from five plasma samples from HIV-1 negative individuals were spiked with virions or mock from Raji-CD4 infected-cells. Then EVs were precipitated by ExoQuick^TM^ from plasmas. The pellet was re-suspended in PBS and laid on iodixanol velocity gradient. RNA was purified and miR-92 (**C**), miR-155 (**D**), and miR-223 (**E**) were amplified by RT-PCR and measured in each gradient fraction. Values are normalized as 38—Ct. Comparisons are based on two-way ANOVA with the Bonferroni post-test. (* *p* < 0.05, ** *p* < 0.01, *** *p* < 0.001). Values are mean ± SEM of at least three independent experiments.

We have reported previously that plasma EVs in ART-naïve HIV-1 patients were rich in miR-92, miR-155, and miR-223 and that the abundance of miR-155 and miR-223 was strongly correlated with EVs size and AChE activity [5]. Deep sequencing revealed that the most abundant species of RNA in EVs is microRNA [46]. To determine if HIV-1 could cause the noted effects on microRNA, we compared expressions by NL4-3 infected and mock-infected Raji-CD4 cells. Although infection with the virus might have increased miR-223 in cells (Figure 6A), only a significant increased in miR-155 EVs has been observed (Figure 6B). Plasmas from HIV-negative subjects were then spiked with the virus from HIV or mock-infected cells before EVs were precipitated by ExoQuick^TM^ and separated on iodixanol velocity gradients (Figure 6C–E). As seen in Figure 6D, miR-155 was detected in iodixanol fractions 9.6–12%, whether the EVs were obtained from infected or mock-infected cells. The differences in abundance in 16.8% and 18% fractions were much more significant (Figure 6D) than for miR-92 (Figure 6C) or miR-223 (Figure 6E). Fractions 10.8% and 18% with virions contained more miR-223, while only the fraction 9.6% contained more miR-92. The otherwise similar distribution of these molecules led us to focus on miR-155, of which the relative abundance in fraction 16.8% appears to be more consistent and to increase after infection with HIV-1.

### 2.3. Iodixanol Velocity Gradient Reveals Elevated AChE Activity and Increased miR-155 Expression in Large EVs in ART-Naïve HIV-1 Patients

**Figure 7:**
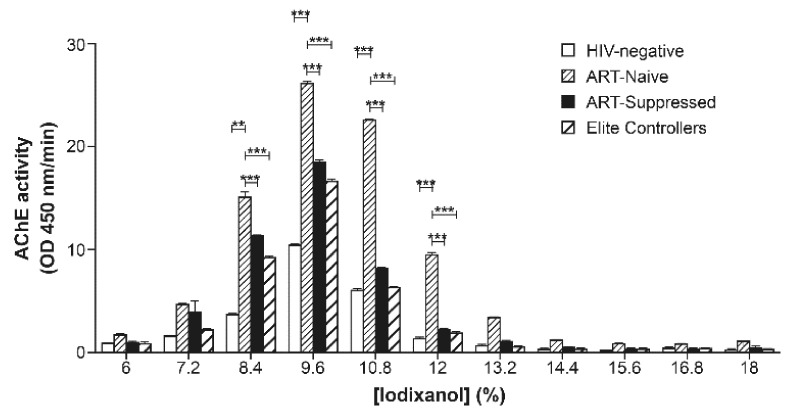
Abundance of EVs in the plasma of HIV-1 patients. Plasmas from each group (HIV-1 negative, ART-naïve, ART-suppressed, and elite controllers) were pooled (*n* = 8), concentrated by ExoQuick^TM^, and resolved by ultracentrifugation on iodixanol velocity gradient. AChE activity was measured in each fraction. One-way ANOVA was used with the Tukey post-test. (** *p* < 0.01, *** *p* < 0.001).

Since the iodixanol velocity gradient offered an optimal resolution of EVs, we undertook the study of plasma EVs from individuals infected with HIV-1. We pooled plasmas of 8 participants for each groups of patients with a different disease status (Table 1) and isolated EVs with ExoQuick^TM^. EVs analysis was next resolved using velocity gradient. Results show AChE activity mostly in fractions 8.4–12% iodixanol for all groups, with peaks in fractions 9.6% and 10.8% of iodixanol (Figure 7).

**Figure 8:**
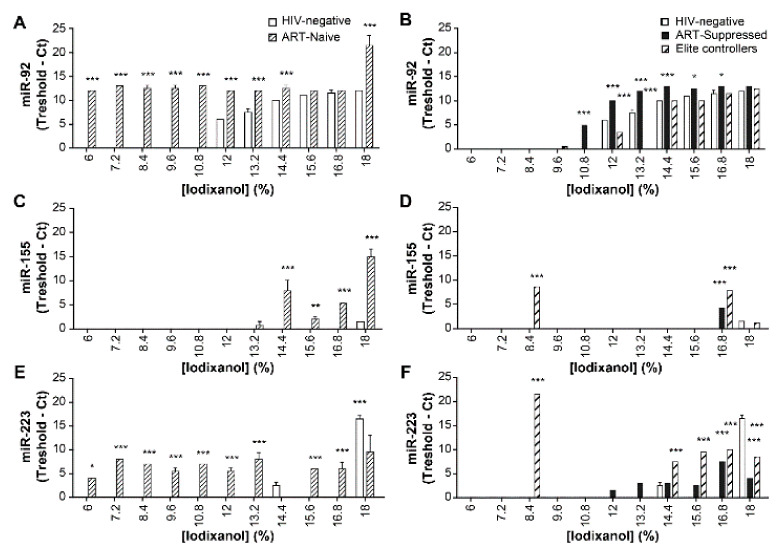
Comparison of HIV-negative subjects and HIV-1 patients in terms of plasma EVs miR-92, miR-155, and miR-223 distributions in the iodixanol velocity gradient. Plasmas were pooled (*n* = 8 for each group) and pre-treated with proteinase K before EVs precipitation with ExoQuick™. MicroRNA in each gradient fraction was amplified by RT-PCR. Values are normalized as 38—Ct. HIV-1 negative versus ART-naïve (**A**,**C**,**E**) and HIV-1 negative versus ART-suppressed and elite controllers (**B**,**D**,**F**) were compared using two-way ANOVA and the Bonferroni post-test. Analyses were repeated at least two times (* *p* < 0.05, ** *p* < 0.01, *** *p* < 0.001).

Therefore, the distribution of plasma EVs bearing miR-92, miR-155, and miR-223 in the iodixanol gradient was examined (Figure 8). Fractions 14.4–18% from ART-naïve patients were found rich in miR-155 (Figure 8C), as were fractions 8.4% and 16.8% from elite controllers and fraction 16.8% from ART-suppressed patients (Figure 8D). In contrast, miR-223 and miR-92 were distributed widely in ART-naïve patients, with an abrupt increase of miR-92 in fraction 18% (Figure 8A). Except in ART-naïve patients, miR-92 was not detected in fractions below 10.8%. MiR-223 was elevated conspicuously in fraction 8.4% from elite controllers (Figure 8F). All infected patient groups presented elevated miR-155 in a fraction of 16.8%. MiR-155 was also concentrated in last fractions in HIV-1-negative plasmas (Figure S3), suggesting that a population of uncharacterized vesicles enriched in miR-155 co-localizes with larger EVs independently of infection. Together these results show that microRNA expression differs in ART-naïve group from other groups. In addition HIV-1 infection increase EVs production (Figure S4A) and calprotectin levels in plasma (Figure S4B,C).

**Figure 9:**
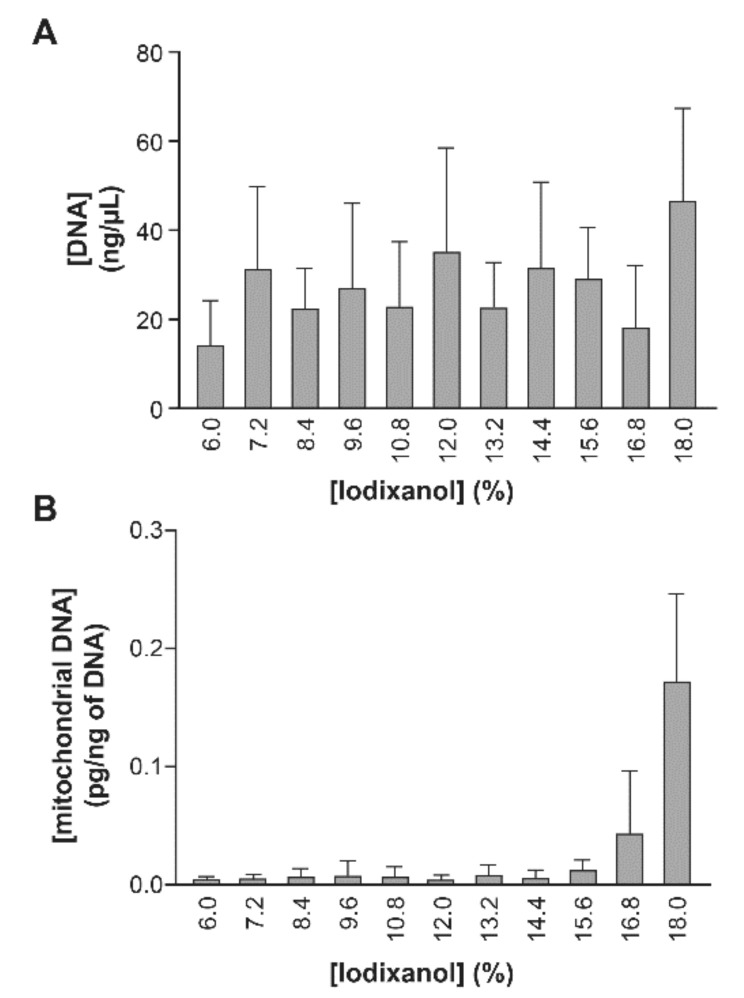
Distribution of EVs-associated mitochondrial DNA in the iodixanol velocity gradient. Cell culture supernatant from uninfected cells was microfiltered then ultra-centrifuged at 100,000× *g* for 45 min. The EVs pellet was re-suspended in PBS. Total DNA was extracted from each fraction of the velocity gradient using the phenol/chloroform method and quantify by Biodrop (**A**). Mitochondrial DNA was quantified by qPCR using a standard curve and expressed as mean ± SEM of the technical triplicates of three independent cells preparation (**B**).

Despite of the presence of DNA (Figure 9) and RNA in every fraction (Figure S5C), coupled with the large variability in EVs size throughout the gradient (Figure S5B), the velocity gradient using a vertical rotor provided the best resolution of the heterogeneous EVs. Furthermore, the velocity gradient separated AChE-positive EVs from other plasma components such as calprotectin (Figure S4) and mitochondrial DNA (Figure 9B).

**Figure 10:**
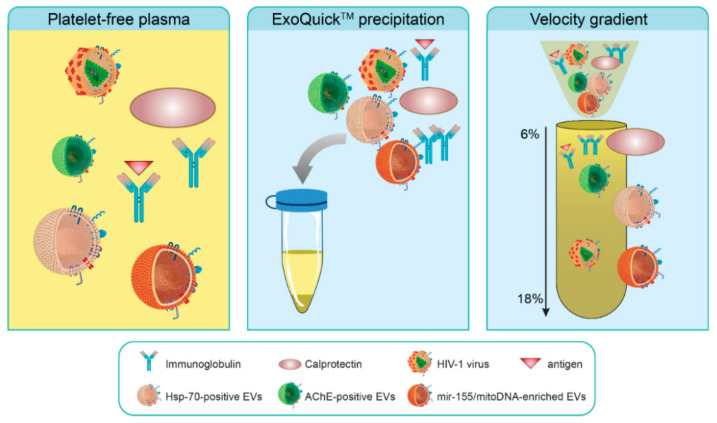
Iodixanol velocity gradient efficiently separates heterogeneous potential biomarkers from the plasma. The left panel represents non-exhaustive potential biomarkers present in the plasma. The middle panel illustrates that ExoQuick™ precipitates several biomarkers along with virions and calprotectin. The right panel illustrates that the velocity gradient might separate calprotectin, AChE-positive and Hsp-70-positive EVs, virions, and possibly new types of EVs enriched in miR-155 and mitochondrial DNA.

Alternatively to gradient separation, ultracentrifugation of plasma followed by ExoQuick™ precipitation, allow to obtain two types of EVs (large and small) (Figures S5A and S6A). Finally, large EVs appear to contain little AChE (Figure S6B), whereas a subgroup of large EVs is rich in miR-155 (Figure S6D) and mtDNA (Figure S6F) and smaller AChE-bearing ones containing increased amounts of miR-92 (Figure S6C). All these results suggest that precipitated heterogeneous plasma components may be separated by a velocity gradient as illustrated in Figure 10.

## 3. Discussion

When studying the effects of viral infections on EVs in biological fluids such as plasma, it is crucial to bear in mind that proteins/protein aggregates or enveloped viruses such as HIV-1 may be challenging to distinguish from vesicles of interest. The effects of HIV-1 infection on EVs production and contents cannot be adequately studied unless the presence and absence of virions can be shown with certainty, in other words, only if virus-free EVs fractions and EVs-free virus fractions can be obtained from cultures of infected cells and the plasma of infected patients. This study showed that the sucrose density gradient or commercial kits are inadequate for this purpose and concentrate materials since virus and EVs are present in the same fractions. In contrast, the velocity gradient more efficiently separated proteins, AChE-positive EVs, Hsp-70-positive EVs, virions, miR-155-enriched EVs, and possibly mitochondrial DNA-enriched EVs, as illustrated in Figure 10.

To date, various kits have been developed and commercialized to purify EVs in a quick and easy steps manner. Comparison of three commercially kits to purify EVs from HIV-1 showed that none of the commercial kits, namely ExoQuick^TM^ or ExoQuick-TC^TM^ from System Biosciences, Exo-spin^TM^ Blood from Cell Guidance Systems and Total Exosome Isolation (from plasma) from Thermo scientific, or either differential centrifugation method by itself discriminates between HIV-1, EVs, and the protein calprotectin.

We also compared the sucrose density gradient and iodixanol density or velocity gradients and examined a broad range of EVs from a cultured human cell-line infected with HIV-1 virus, plasmas from HIV-1-negative individuals in the presence or absence of HIV-1 virus, or plasmas from HIV-1 patients. Our results showed that these particles and proteins could be better separated on an iodixanol velocity gradient once centrifugation time and iodixanol concentration are optimized. AChE activity is found typically in fractions 8.4–12% iodixanol velocity gradient [9,37,44,47,48,49]. It should be noted that these plasma samples were frozen, but this had no apparent impact on the AChE activity profile in the gradient (data not shown). The conspicuous increase of AChE activity in ART-naïve patients corroborates our previous findings [5].

Transport proteins, microRNA, and mRNA in EVs participate in intercellular communication and cell transformations [7] and might act as markers of disease [5]. Evidence is accumulating that their composition changes with disease stages and that they might play significant roles in infection and pathogenesis. In the present study, an iodixanol-based centrifugal velocity gradient was used to improve the resolution of vesicles suspended in the plasma of HIV-1 patients. The significant finding revealed the variability of plasma vesicles and their microRNA contents. The most interesting results were the variation of miR-155 levels in a hitherto uncharacterized EVs fraction also rich in mitochondrial DNA. Our results also suggest that miR-155-rich EVs come from mitochondria and that this EVs secretion could correlate with decreased numbers of mitochondria in PBMCs of HIV-1 patients and the resistance of infected cells to apoptosis. The microRNA miR-155 is known to be expressed inside mitochondria (and is also called mitoMir) [50,51,52].

We have shown that ExoQuick™ precipitation does not yield virus-free EV preparations, in contrast with the ultracentrifugation on velocity gradient. The unexpected result was that RNA and microRNA were not concentrated in the EVs-containing fractions but rather were present in all fractions, while calprotectin was concentrated in the first fractions, corresponding to the free proteins.

Characterization of plasma EVs is made more complicated by their specific biogenic mechanisms and cellular origins that may alter their profile. Although microvesicles, EVs and apoptotic bodies constitute most of the EVs in plasma, their distribution can be far more heterogeneous. Different pathways and corresponding subpopulations have been described both for EVs biogenesis and microvesicle secretion [53]. We detected Hsp-70 and Alix in different fractions of our velocity gradient. Moreover, HLA-DR is detected in the same fractions of Alix, Hsp-70, and virions, suggesting an increased production of plasma EV-HLA-DR+ during HIV-1 infection. Our analyses also revealed pronounced differences between groups and between patients in plasma EVs size distribution throughout the velocity gradient. Since we have chosen a limited number of EV’s markers, it is clear that some EV populations are missing in our analysis.

Targets of miR-155 on host mRNA and HIV-1 mRNA are numerous [54]. This microRNA is involved in both innate and adaptive responses. The amount of EV-associated miR-155 increased, as was the case for infected Raji-CD4 cells with NL4-3 (Figure 6B,D). These results are consistent with HIV-1 infection modulating the microRNA profile of lymphocytes as reported previously [55,56] and show for the first time an increase of miR-155 in EVs from HIV-1-infected cells, concentrating these EVs in the denser iodixanol fractions.

## 4. Materials and Methods

### Reagents and Antibodies

Reagents: G418 was purchased from InvivoGen (San Diego, CA, USA). RPMI 1640, high-glucose DMEM, penicillin G, proteinase K, streptomycin, l-glutamine, and bovine serum albumin were purchased from Wisent Inc. (Saint-Bruno, QC, Canada). Acetylthiocholine, 5,5-dithio-bis(2-nitrobenzoic acid), 2,2′-dithiodipyridine (AT-2), Optiprep™ (60% iodixanol), trichloroacetic acid, sodium orthovanadate (Na_3_VO_4_), Tween-20 and fetal bovine serum (FBS) were all purchased from Sigma-Aldrich (St. Louis, MO, USA). Luminata forte was obtained from Millipore Corporation (Bedford, MA, USA). ExoQuick^TM^ and ExoQuick-TC^TM^ were purchased from System Biosciences (Palo Alto, CA, USA) via Cedarlane (Burlington, ON, Canada). Aprotinin and leupeptin were purchased from Boehringer (Burlington, ON, Canada). BCA protein assay kit was purchased from Thermo Scientific (Rockford, IL, USA). Peroxidase colorimetric substrate (TMB) was purchase from Neogen Corporation (Lexington, KY, USA).

Antibodies: Anti-HLA-DRα (DA6.147), anti-ICAM-1 (G-5, cat. no. sc-8439), anti-Lamp2 (H4B4, cat. no. sc-18822), anti-Hsp-70/Hsc-70 (W27, cat. no. sc-24), and anti-Alix (349, cat. no. sc-53538) were purchased from Santa Cruz Biotechnologies (Santa Cruz, CA, USA). Anti-Tsg-101 (4A10, cat. no. ab83) was obtained from Abcam (Toronto, ON, Canada). Horseradish-peroxidase-(HRP), labeled donkey anti-mouse IgG F(ab’)2 fragment (cat. no. 715-036-150) and HRP-labeled goat anti-mouse IgG antibody (cat. no. 115-035-062) were purchased from Jackson ImmunoResearch Laboratories (West Grove, PA, USA) and were used for western blots and calprotectin ELISA, respectively. Monoclonal antibody specific for human calprotectin (mAb 27E10) was purchased from Hycult^®^ Biotech (Plymouth Meeting, PA, USA). Anti-p24 and anti-p24 biotin-conjugated antibodies, respectively from hybridomas 183-H12-5C (capture antibody) and 31-90-25 (detection antibody), were used for enzyme-linked immune-sorbent assay (ELISA) of HIV-1 protein p24 and obtained through the AIDS Repository Reagent Program (Germantown, MD, USA). Streptavidin-PolyHRP40 was purchased from Fitzgerald Industries (Burlington, ON, Canada). Anti-p24 was purified using mAbTrap protein affinity columns according to the manufacturer’s instructions (Pharmacia Technology AB, Uppsala, Sweden).

Studied population: This study received approval from the ethics review boards of the Centre de recherche du CHU de Québec (Quebec City, QC, Canada) and McGill University Health Centre (Montreal, QC, Canada) (C12-03-167). All subjects were anonymous volunteers and provided written informed consent. A total of 28 HIV-1-infected patients recruited at McGill University participated in the study (Table 1): 9 chronically infected patients having never received antiretroviral therapy (ART-naïve), 10 receiving an anti-retroviral therapy and having an undetectable viral load (≤50 copies per mL of plasma) (ART-Suppressed), and 9 who spontaneously controlled viral replication without treatment for over 7 years (Elite controllers). Nine uninfected control subjects also participated (HIV-1 negative). Samples (26) were also obtained from a cohort of patients infected with HIV-1, from “Unité Hospitalière de Recherche, d’Enseignement et de Soins du SIDA/VIH/hépatite (UHRESS) du CHUL, CHU de Québec, Université Laval”. Blood was collected in vacuum tubes containing EDTA or citrate as an anticoagulant and centrifuged at 400× *g* for 10 min. The plasma supernatant was centrifuged at 3000× *g* for 10 min to obtain platelet-free plasma, which was aliquoted and stored at −80 °C until analysis.

Virus production: HIV-1 was produced first by transfection of HEK 293T cells as described previously [57] with X4-tropic clone pNL4-3 provided by the AIDS Repository Reagent Program, Germantown, MD. The NL4-3, thus obtained, was used to infect Raji-CD4 cells or Raji-CD4 DCIR cells, a human lymphoblastoid line derived from a Burkitt lymphoma and modified to express the CD4 receptor only or co-expressing CD4 and DCIR receptors [58]. Infection was carried out in suspension for 2 h with 25 ng of p24 per 10^6^ cells. Mock infection with inert particles at the same concentration was carried out in parallel. Raji-CD4 cell-lines were washed and then maintained in culture at 2 × 10^5^/mL at 37 °C in RPMI containing penicillin G and streptomycin (100 U/mL each) and supplemented with fetal bovine serum (10%). The medium was added every 2 days. On day 5, the culture supernatant was microfiltered on the cellulose membrane (0.22 µm pore, Millipore, Billerica, MA, USA) then centrifuged at 100,000× *g* for 45 min at 4 °C (Optima L-90K Beckman Coulter, 70Ti rotor, Fullerton, CA, USA). The pellet was washed (except for Figure 1, Figures S1 and S2) and resuspended in PBS (300 µL) before precipitation with ExoQuick-TC^TM^ as recommended by the manufacturer or resuspended in PBS (50 µL/3 × 10^6^ cells) before directly loading on sucrose density gradient, iodixanol density gradient, or iodixanol velocity gradient. An in-house double-antibody sandwich enzyme-linked immunosorbent assay (ELISA) specific for viral protein p24 [59] was used to standardize virus titer at both stages (HEK 293T and Raji-CD4 culture). All fetal bovine serum was centrifuged at 120,000× *g* for 18 h to remove as many EVs as possible [60,61].

Purification of EVs from plasma: Purification of EVs was done as described previously [5,6]. Blood was obtained by venipuncture with citrate or EDTA as an anticoagulant. Blood was centrifuged for 10 min at 400× *g* and the platelet were then removed by centrifugation for 10 min at 3000× *g* at room temperature to obtain platelet-poor plasma (PPP), which was stored at −80 °C. Thawed PPP was centrifuged for 10 min at 3000× *g* to obtain platelet-free plasma (PFP). Plasma (250 µL) was treated or not with proteinase K (1.25 mg/mL, Ambion™, Thermo Fisher Scientific, Waltham, MA, USA) for 10 min at 37 °C. EVs were concentrated by mixing 250 µL of plasma with 63 µL of ExoQuick^TM^ and incubated for 30 min at 4 °C before centrifugation at 1500× *g* for an additional 30 min. The pellet was washed and resuspended in PBS at a final volume of 250 µL. Using the Exo-spin^TM^ Blood method (Cell Guidance Systems, St. Louis, MO, USA), EVs purification comprised of gently mixing 500 μL of plasma with 250 μL of Buffer A, holding for 30 min at 4 °C and centrifuging at 20,000× *g* for 30 min. The precipitate was resuspended in 100 μL of Buffer B (PBS) and processed through the Exo-spin^TM^ column. The final volume of the eluted fraction was completed to 250 μL. Purification using the Total Exosomes Isolation kit (Thermo Scientific, Rockford, IL, USA) comprised treating 250 µL of plasma with 50 μL of proteinase K solution (optional, indicated in the Legends), adding 150 μL of precipitation reagent, holding at room temperature for 10 min, centrifuged for 5 min at 10,000× *g*, resuspending the precipitate in 250 μL of microfiltered PBS.

Recovery of EVs from plasma spiked with HIV-1 or with a mock: Plasmas from five HIV-1 negative participants were pooled with NL4-3 produced on Raji-CD4 cells (3.2 µg of p24 per mL) or an equivalent volume of mock virus suspension. EVs were then concentrated using the ExoQuick^TM^ procedure mentioned above and loaded onto the density or velocity gradient.

Sucrose density gradient: The sucrose density gradient protocol was adapted from a method described previously [62]. Samples were placed at the bottom of the tube, and 2 mL of 2.5 M sucrose solution (pH 7.4) was added gently to minimize mixing. The 0.515–2.0 M gradient was then produced by overlaying 750 µL for each concentration increment. The tubes were centrifuged in a swinging bucket rotor (SW40, Figure 4) or SW41 (Figure S1) overnight at 200,000× *g* (4 °C), the Optima L-90K Beckman Coulter centrifuge. Fractions (1 mL) were collected from the top of the gradient for analysis. The density of each fraction was assessed after ultracentrifugation of fetal bovine serum’s EVs in both rotors and measured with a refractometer. The density of each fraction represents the mean of two different gradients fractions.

Iodixanol density gradient: A discontinuous gradient of iodixanol diluted in 0.25 M of sucrose was created by layering 3 mL each of 40%, 20%, 10% iodixanol and 2.3 mL of 5% iodixanol. EVs samples (300–350 µL) were layered onto the 5% portion and were centrifuged at 100,000× *g* for 18 h at 4 °C in an SW41 swinging rotor (Beckman Coulter). Twelve fractions (1 mL each) were collected from the top and analyzed as described previously [63]. Where indicated, proteins were measured in each fraction using the Thermo scientific Pierce BCA protein assay kit as per the manufacturer’s instructions.

Iodixanol velocity gradient: The iodixanol velocity gradient protocol has been described previously [37]. Samples were layered onto a 6–18% iodixanol continuous gradient and centrifuged at 180,000× *g* for 50 min (or 75 min, Figure 1D,E and Figure 2A) in a Stepsaver 65V13 vertical rotor (Fisher Scientific) or NVT65 vertical rotor (Beckman). The fractions (900 μL) were collected from the top of the liquid column. Where indicated, proteins were measured in each fraction using the BCA protein assay kit as per the manufacturer’s instructions.

Acetylcholinesterase activity: AChE activity was assayed as least technical duplicate. The final re-suspension (100 µL) containing 125 mM acetylthiocholine and 0.1 mM 5,5-dithio-bis(2-nitrobenzoic acid) in PBS was held at room temperature [5]. Changes in absorption were monitored at 450 nm for 10 min with a plate reader spectrophotometer SPECTRAmax 190, Molecular Devices, San Jose, CA, USA.

Calprotectin ELISA: Calprotectin in plasma was measured using an in-house ELISA [64] based on the sandwich principle. As described previously, the standard was purified from human neutrophil cytosol [65]. The volumes added were 100 μL unless otherwise indicated. The 96-wells were coated overnight at 4 °C with polyclonal rabbit anti-human S100A9 IgG diluted to 1.25 µg/mL in 0.1 M NaHCO_3_ buffer pH 9.6 as previously reported in Tessier et al. [64]. The following procedure was then performed at room temperature: Plates were washed three times with PBS/0.1% Tween 20 (wash buffer) and blocked for 1 h with 200 μL of PBS/0.1% Tween 20/2% bovine serum albumin (blocking buffer). Samples and standard diluted in blocking buffer were added, followed by 25 µL of lysis buffer (PBS, 2.5% Triton X-100, 1% trypan blue, 0.05% Tween 20). Plates were left for one hour then washed three times with wash buffer. Primary antibody (mouse anti-human S100A8/A9 monoclonal antibody clone 27E10, 0.075 μg/mL, Hycult^®^ Biotech) diluted in blocking buffer was then added. After 1 h, plates were washed three times, and a secondary antibody (HRP-conjugated goat anti-mouse IgG diluted 1/10,000 in blocking buffer) was added. After one hour, plates were rewashed three times, and the peroxidase colorimetric substrate (TMB) was added. The reaction was stopped by adding 100 μL of 0.18 M H_2_SO_4_. Absorbance at 450 nm was measured using an ELX808 plate reader (BIO-TEK Instruments, Winooski, VT, USA). Data were acquired and processed using KC4 software and a 4-parameter logistic non-linear regression model. The calprotectin detection range was 0.1–100 ng/mL.

EVs size measurement: EVs hydrodynamic size was measured using dynamic light scattering (DLS). For each sample, two measurements were performed at 4 °C using a Zetasizer NanoS (Malvern Instruments, Ltd., Malvern, UK). Values presented in this study are the average of two measurements per sample.

SDS-PAGE and western blot analyses: Proteins were precipitated by adding 49 µL of 100% TCA to 280 µL of gradient fraction (final TCA concentration 15%) or acid methanol 0.01 M HCl (Figure 1 and Figure S1). After TCA precipitation, fractions were stored at −80 °C for 24 h, then thawed and centrifuged at 15,000× *g* for 10 min at 4 °C. The supernatant was discarded. The pellet was washed twice with absolute ethanol. The dried pellet was resuspended in 35 µL of lysis buffer (1% NP40, 20 mM Tris-HCl pH 7.5, 137 mM NaCl, 1 mM EDTA, 2 mM orthovanadate, 10 µg/mL of aprotinin, 10 µg/mL of leupeptin, 2 mM phenylmethylsulfonyl fluoride, and 50 µg/mL of soybean trypsin inhibitor), mixed with 35 µL of reducing modified Laemmli’s sample buffer (2×, held at 100 °C for 5 min and then stored at −20 °C until analysis [66]. Proteins in sample buffer (8 µL) were then subjected to 10% SDS polyacrylamide gel electrophoresis and transferred to the PVDF membrane (Millipore Corporation, Bedford, MA, USA). Before incubation with primary and secondary antibodies, membranes were blocked for 30 min with 5% non-fat skim milk dissolved in Tris-buffered saline with 0.1% Tween 20. Immunoblotting with primary antibodies was carried out overnight at 4 °C. Anti-Alix, anti-Hsp-70, and anti-Tsg-101 were diluted 1/100, anti-HLA-DR (1 mg/mL), and anti-p24 (1 mg/mL) 1/500. Membranes were washed three times with Tris-buffered saline containing 0.1% Tween 20 (or 0.05% for anti-Tsg-101 and anti-HLA-DR) held for 45 min HRP-conjugated secondary antibody diluted 1/10,000, rewashed three times, and developed with the Luminata forte HRP substrate.

MicroRNA quantitation: Raji-CD4 cells (1 × 10^6^) were washed twice with PBS and resuspended in TRIzol (Thermo scientific, Rockford, IL, USA). EVs in PBS were diluted in 3 volumes of TRIzol LS (Thermo scientific, Rockford, IL, USA). Samples were stored at −80 °C until analysis. Total extracted RNA was measured using a BioDrop device (MBI equipment, CA, USA). The treatment consisted of 1 µg (from cells) or 400 ng (from EVs) with DNase according to the manufacturer’s instructions (Thermo scientific, Waltham, MA, USA). Reverse transcription was performed using a HiFlex miScript RT II kit (Qiagen, Hilden, Germany). Mature miR-155 (#MS00031486), miR-223 (#MS00003871), and miR-92 (#MS00006594) were quantitated as cDNA by real-time PCR (RT-qPCR) using a miScript Primer Assay kit and miScript SYBR Green PCR kit (Qiagen, Hiden, Germany). Amplification was performed in a CFX Connect real-time PCR Detection System (BIO-RAD, Hercules, CA, USA) using 40 cycles of 94 °C for 15 s, 55 °C for 30 s, and 72 °C for 30 s. Reaction specificity was confirmed using the melt curve procedure (65–95 °C, 0.5 °C per 5 s) at the end of the amplification protocol according to the manufacturer’s instructions. A detection threshold cycle (Ct) of 38 was considered negative and used as the detection reference. Absolute quantification was based on standard curves with synthetic miRNAs as previously described [6].

DNA extraction: Culture supernatants from Raji-CD4 cell-lines were microfiltered (0.22 µm), distributed in Beckman–Coulter tubes (Cat. 355618), and ultracentrifuged at 100,000× *g* for 45 min. The EVs pellets were resuspended in PBS and stored at −80°C until analysis. Samples were layered onto the velocity gradient and centrifuged as described above. To 200 µL of each fraction, 480 µL of lysis buffer (0.4% *m*/*v* Tris base, 1% *m*/*v* sodium dodecyl sulfate, and 100 mM EDTA), and 20 µL of proteinase K (25 mg/mL) were added, followed by holding at 55 °C for 10 min, adding 500 µL of phenol/chloroform/iosamyl alcohol, and centrifugated at 12,000× *g* for 3 min at 4 °C. The aqueous phase was mixed with an equivalent volume of chloroform, centrifuged again, mixed with absolute ice-cold ethanol, NaCl solution (to 0.5 M final), and 1 µL of GlycoBlue Coprecipitant, held at −80 °C for 30 min, and centrifuged at 12,000× *g* for 15 min. The DNA pellets were washed with cold 70% ethanol and quantified using a BioDrop spectrophotometer.

Quantitative PCR: Mitochondrial DNA in 2 ng of total DNA was measured by quantitative PCR with a SYBR^®^ Green PCR kit (Qiagen, Hilden, Germany) and specific primers. Forward: ACGCCTGAGCCCTATCTATTA-3′ (Tm = 54.9), reverse: 5′-GTTGACCTGTTAGGGTGAGA AG-3′ (Tm = 55). The amplification program was 15 min at 95 °C (enzyme activation) then 40 cycles of 15 s at 94 °C (denaturation), 55 °C for 30 s (annealing), and 72 °C for 30 s (elongation).

Statistical analysis: Data were presented as mean or median ± standard error or median interquartile range. Comparisons between groups were based on two-way ANOVA and the Bonferroni post-test. HIV-1 viral loads were compared between groups using single-factor ANOVA, followed by Tukey’s post-hoc test for multiple comparisons. The Wilcoxon matched-pairs signed-rank test was used (Figure S6). All statistical analyses were performed using GraphPad Prism 5 software. The threshold *p*-value for declaring to be significant was <0.05. Asterisks denote the degree of significance (* *p* < 0.05, ** *p* < 0.01, and *** *p* < 0.001).

## 5. Conclusions

This study showed that miR-155 increases in plasma EVs in HIV-1 patients. Its presence in less characterized velocity gradient fractions was novel and needs to be investigated using lipidomic, proteomic, and cytofluorometric analyses to determine the cellular and intracellular compartments of origin. This analysis may prove crucial to improving our understanding of the role of miR-155 in HIV-1 pathogenesis. Once the physiological functions and biological significance of EVs are more understood, their profiling could provide to be handy biomedicine tools and lead to innovative research and development. This study shows that velocity gradient centrifugation likely remains the most effective method of resolving EVs populations.

## Figures and Tables

**Table 1:** Clinical characteristics of participants.

	HIV-Negative Subjects			HIV-1-Positive Subjects								
				ART-Naive			ART-Suppressed			Elite Controllers		
	*n* = 9			*n* = 9			*n* = 10			*n* = 9		
Viral load (log_10_ copies/mL)		NA		3.97	±	1.074	<1.7			<1.7		
Years of infection		NA		2.11	±	1.16	8.50	±	3.44 b***	12.33	±	3.44b ***c*
CD4 (cells/μL)	867.1	±	186.0	511.6	±	299.1 a*	710.9	±	259.9	652.1	±	277.8
CD8 (cells/μL)	420.1	±	127.1	898.0	±	387.3 a**	795.0	±	227.6 a*	562.8	±	255.0
CD4/CD8 ratio	2.17	±	0.56	0.68	±	0.52 a***	0.93	±	0.32 a***	1.38	±	0.61 a*b*
Age (years)	40.78	±	8.37	34.00	±	7.95	47.3	±	9.94 b**	48.44	±	7.53 b**

Clinical characteristics of patients. All clinical parameters are reported as mean ± SD. ^a^ *p* value vs. healthy subjects; ^b^ *p* value vs. ART-naive subjects; ^c^ *p* value vs. ART-suppressed subjects; * *p* < 0.05; ** *p* < 0.01; *** *p* < 0.001 (ANOVA, Tukey post-test), NA: not applicable.

## Data Availability

The data presented in this study are available in the manuscript main tables and supplementary tables.

## Supplementary Materials

The following are available online at https://www.mdpi.com/article/10.3390/pathogens10050526/s1, Figure S1: ExoQuick-TC^TM^ and sucrose gradient appears not to separate EVs from the virus. Figure S2: Quantification of HIV protein p24 in EVs precipitated using a commercial kit. Figure S3: Variability of miR-155 distribution in iodixanol velocity gradient fractions of plasma obtained from five HIV-1 negative individuals. Figure S4: Separation of plasma calprotectin and AChE EVs by iodixanol velocity gradient. Figure S5: Size and sedimentation of plasma EVs obtained from two HIV-1 patients. Figure S6: Large EVs are enriched in miR-155 and mitochondrial DNA.
