# Supplementary material for: Velocity Gradient Separation Reveals a New Extracellular Vesicle Population Enriched in miR-155 and Mitochondrial DNA
Source: Pathogens. 2021 Apr 27;10(5):526. doi: 10.3390/pathogens10050526 (PMC8146806; doi:10.3390/pathogens10050526)
Supplement: Supplementary file 1 [file pathogens-10-00526-s001.zip › pathogens-1152550-supplementary.pdf]

Supplementary FIGURES

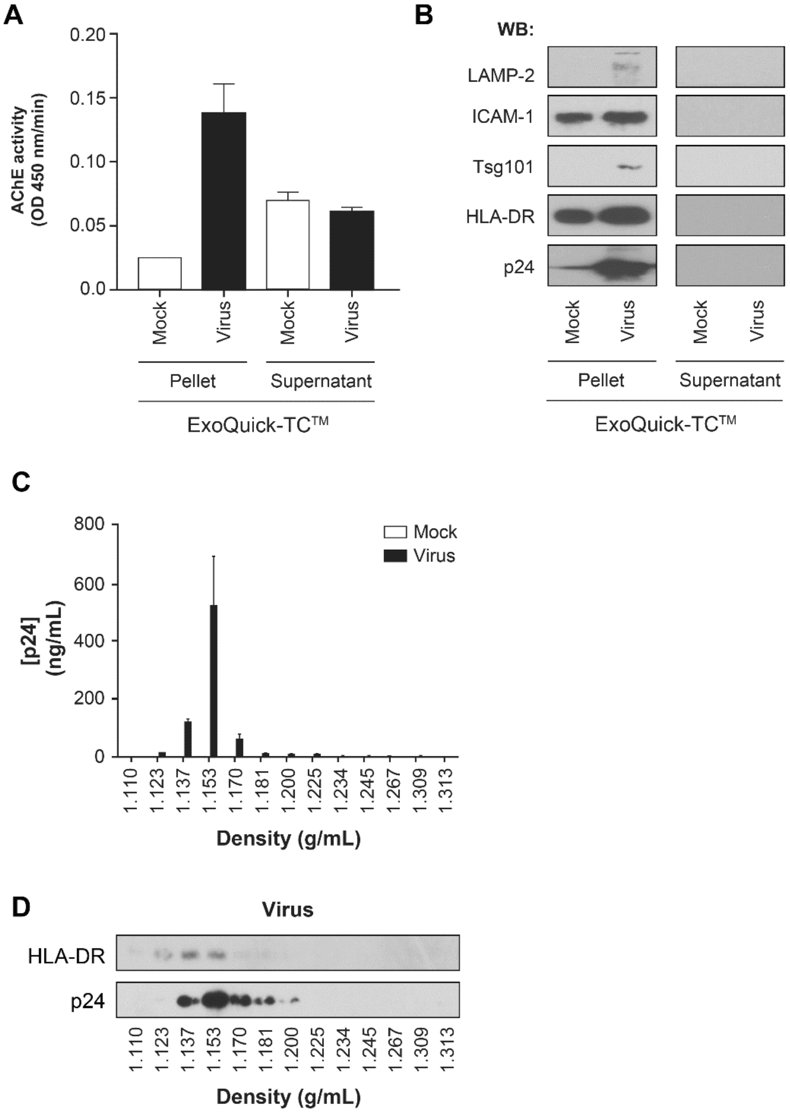

Figure S1: ExoQuick-TC^{TM} and sucrose gradient appears not to separate EVs from the virus. Raji-CD4 DCIR cells were cultured with NL4-3 virus or mock for five days. Microfiltered supernatant was centrifuged at 100,000 x *g* for 45 min. The pellet was re-suspended in PBS before precipitation with ExoQuick-TC^{TM} according to the supplier's instructions **(A, B)**. The presence of EVs, host and viral proteins were assessed by AChE activity **(A)** and western blots **(B)**. 100,000 x *g* pellet containing EVs and virus or mock was recovered from Raji-CD4 infected-cells and was laid on a sucrose gradient (**C, D**). Virions were quantified by p24 ELISA **(C)** and the presence of EVs, host and viral proteins were assessed by western blots **(D)**.

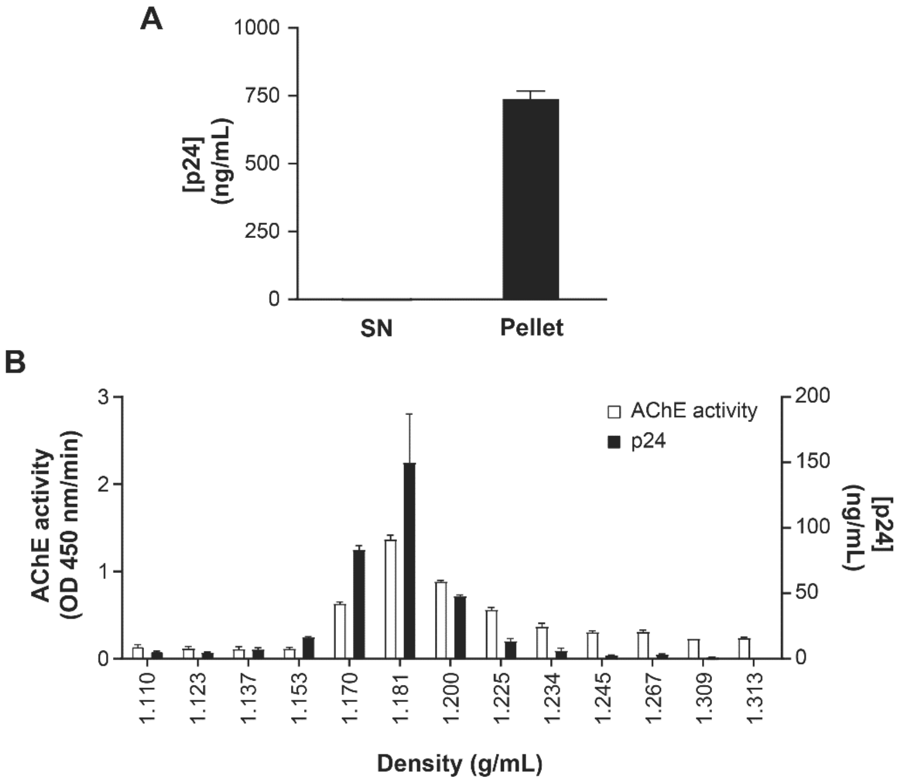

Figure S2: Quantification of HIV protein p24 in EVs precipitated using a commercial kit. A pool from five plasma samples from HIV-1 negative individuals were spiked with virions or mock from Raji-CD4 infected-cells. EVs were then precipitated using ExoQuick^{TM}, and **(A)** the associated p24 concentration was determined by ELISA in the ExoQuick pellet as well as in the supernatant (SN). ExoQuick^{TM} precipitate was centrifuged on a sucrose density gradient before p24 concentration and AChE activity were quantified **(B)**.

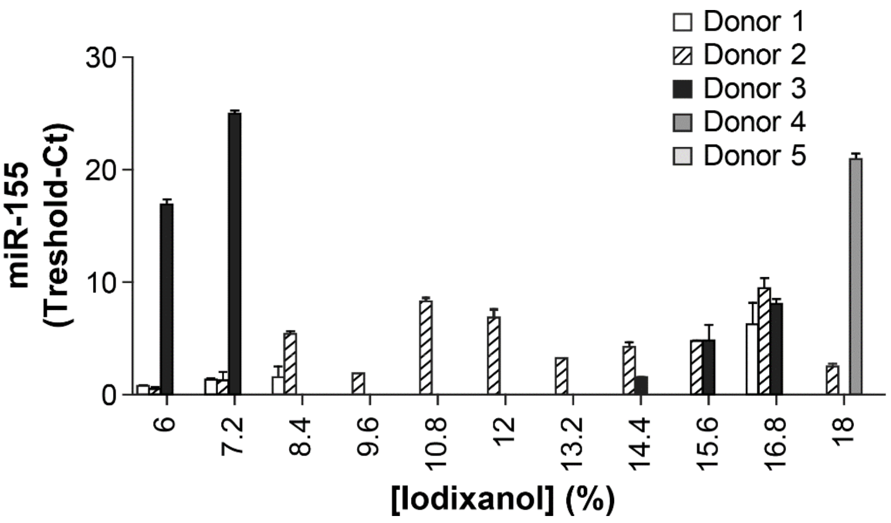

Figure S3: Variability of miR-155 distribution in iodixanol velocity gradient fractions of plasma obtained from five HIV-1 negative individuals. Plasmas (n=5) were processed with ExoQuick^{TM} according to the manufacturer's instructions. EVs precipitate was resolved on the iodixanol gradient, and RNA was extracted from each fraction using Trizol. MicroRNA-155 was amplified by RT-PCR. These results show the variability of miR-155 expression in EVs classes among HIV-1 negative individuals.

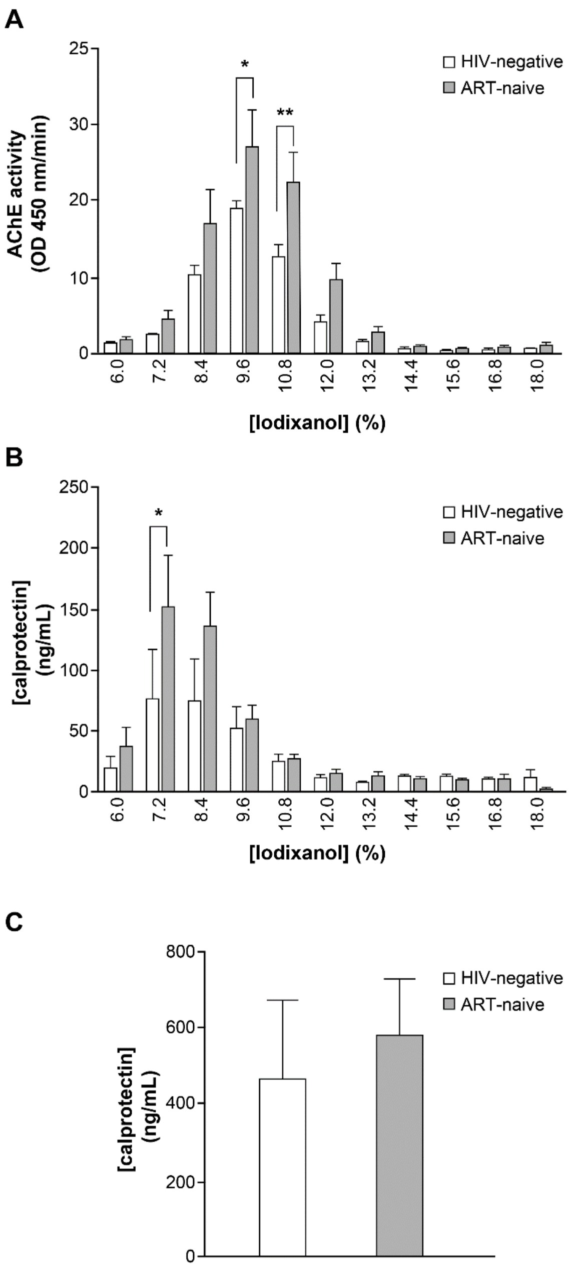

Figure S4: Separation of plasma calprotectin and AChE EVs by iodixanol velocity gradient. The plasma pools from HIV-1 negative individuals (n=3) and ART-naïve patients (n=3) were precipitated by ExoQuick™ and overlaid on iodixanol velocity gradient. The relative abundance of AChE-positive EVs **(A)** and calprotectin **(B)** in the gradient fractions were measured respectively by enzymatic activity and ELISA. The total amount of precipitated calprotectin from plasma was evaluated by ELISA **(C)**. Values shown correspond to one representative experiment of the pool of three donors and are expressed as mean ± SD of technical replicate (* $p < 0.05$, ** $p < 0.01$).

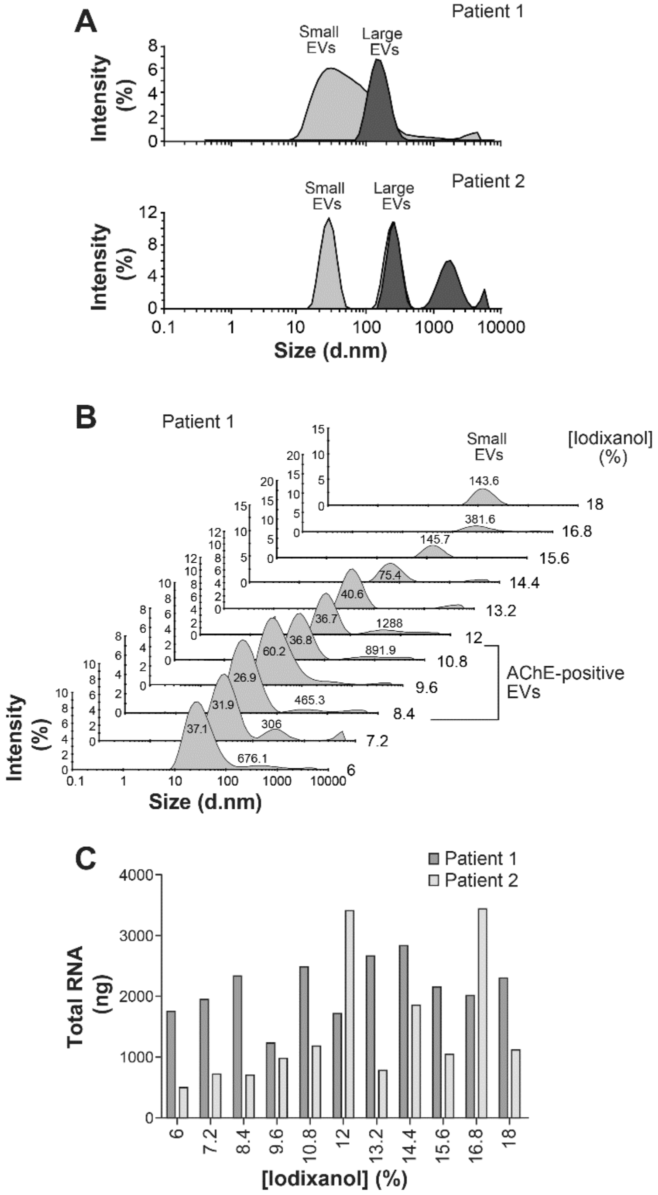

Figure S5: Size and sedimentation behaviour of plasma EVs obtained from two HIV-1 patients. Large EVs in plasma were obtained after 17, 000 x *g* centrifugation, then small EVs in the supernatant were precipitated by using ExoQuick^{TM} according to the manufacturer's instructions. The size **(A)** of each population of EVs was measured by DLS analysis. ExoQuick^{TM} precipitation of small EVs was overlaid on iodixanol velocity gradient and size **(B)** or total RNA **(C)** of small EVs in each gradient fraction was measured by DLS and Biodrop respectively.

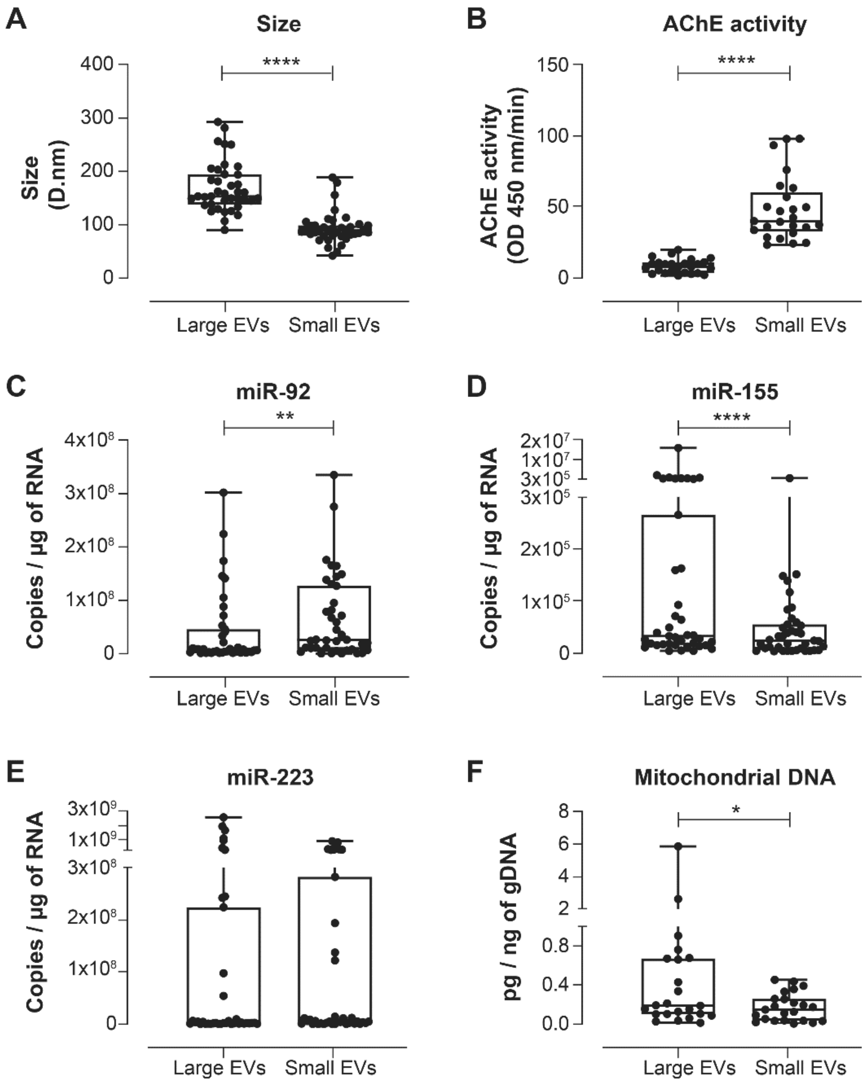

Figure S6: Large EVs are enriched in miR-155 and mitochondrial DNA

Large and small EVs were recovered from platelet-free plasma of HIV-negative subjects after ultracentrifugation at 17, 000 x *g* and ExoQuick^{TM} precipitation as described in Material and Methods. EVs sizing by DLS using the Nanosizer (n = 39) **(A)**; AChE EVs activity (n = 26) **(B)**, mature microRNA in total extracted RNA (n = 39) miR-92 **(C)**, miR-155 **(D)** and miR-223 **(E)**, and mitochondrial DNA level (n = 23) **(F)** were measured by Nanosizer (DLS), enzymatic activity or by RT-qPCR. P values for statistically significant differences are based on the Wilcoxon matched pairs signed rank test (* ≤ 0.05, ** ≤ 0.01, **** ≤ 0.0001).
